# Coral holobiont cues prime *Endozoicomonas* for a symbiotic lifestyle

**DOI:** 10.1038/s41396-022-01226-7

**Published:** 2022-04-20

**Authors:** Claudia Pogoreutz, Clinton A. Oakley, Nils Rädecker, Anny Cárdenas, Gabriela Perna, Nan Xiang, Lifeng Peng, Simon K. Davy, David K. Ngugi, Christian R. Voolstra

**Affiliations:** 1 Department of Biology, University of Konstanz, 78457 Konstanz, Germany; 2 Red Sea Research Center, Biological, Environmental, Science and Engineering, King Abdullah University of Science and Technology (KAUST), 23955 Thuwal, Saudi Arabia; 3 School of Biological Sciences, Victoria University of Wellington, 6012 Wellington, New Zealand; 4 Laboratory for Biological Geochemistry, School of Architecture, Civil and Environmental Engineering, École Polytechnique Fédérale de Lausanne (EPFL), 1015 Lausanne, Switzerland; 5 Alfred-Wegener Institute, Helmholtz Centre for Polar and Marine Research, 27570 Bremerhaven, Germany; 6 Marine Ecology Department, Faculty of Biology and Chemistry, University of Bremen, 28359 Bremen, Germany; 7 Leibniz Institute DSMZ—German Collection of Microorganisms and Cell Cultures GmbH, 38124 Braunschweig, Germany; 8 Present Address: Laboratory for Biological Geochemistry, School of Architecture, Civil and Environmental Engineering, École Polytechnique Fédérale de Lausanne (EPFL), 1015 Lausanne, Switzerland

**Keywords:** Symbiosis, Transcriptomics, Proteomics, Metabolism, Microbial ecology

## Abstract

*Endozoicomonas* are prevalent, abundant bacterial associates of marine animals, including corals. Their role in holobiont health and functioning, however, remains poorly understood. To identify potential interactions within the coral holobiont, we characterized the novel isolate *Endozoicomonas marisrubri* sp. nov. 6c and assessed its transcriptomic and proteomic response to tissue extracts of its native host, the Red Sea coral *Acropora humilis*. We show that coral tissue extracts stimulated differential expression of genes putatively involved in symbiosis establishment via the modulation of the host immune response by *E. marisrubri* 6c, such as genes for flagellar assembly, ankyrins, ephrins, and serpins. Proteome analyses revealed that *E. marisrubri* 6c upregulated vitamin B1 and B6 biosynthesis and glycolytic processes in response to holobiont cues. Our results suggest that the priming of *Endozoicomonas* for a symbiotic lifestyle involves the modulation of host immunity and the exchange of essential metabolites with other holobiont members. Consequently, *Endozoicomonas* may play an important role in holobiont nutrient cycling and may therefore contribute to coral health, acclimatization, and adaptation.

## Introduction

Global change is reshaping marine ecosystems at an unprecedented rate [1–3]. In order to survive, species are forced to migrate, acclimatize, or adapt [3, 4]. Genetic adaptation is slow in organisms with long generation times, such as corals [5, 6]. However, there may be other opportunities for adaptation *sensu lato* beyond genetic adaptation, including the potential for rapid adaptation through changes in the functions and dynamics of host-microbe interactions [5–7]. In numerous host-microbe systems, bacteria aid holobiont health and functioning via structuring of the microbiome [8–10], provisioning of (essential) metabolites or nutrients [11–13] mitigating stress responses [14, 15], or changes in their host’s life history [16]. Bacteria in corals are thought to support holobiont functioning via nutrient cycling [17–22], antimicrobial activity [23, 24], and antioxidant capacity [25].

*Endozoicomonas* have emerged as prevalent microbiome members throughout a range of tropical corals [9, 13, 26]. They are often abundant in the tissues of healthy corals, but exhibit greatly reduced relative abundances in stressed, diseased, and bleached corals as well as, and in corals on degraded reefs [27–30] (but see also ref. [31]). Consequently, it has been proposed that *Endozoicomonas* may be beneficial for the health and functioning of coral holobionts, e.g. via DMSP transformation [13, 32, 33] or amino acid and carbohydrate metabolism [9, 13]. Importantly though, while genetic features such as repeats and pseudogenization suggest a spectrum of “host-restrictedness” of some cultured *Endozoicomonas* isolates [14], their relatively large genome sizes indicate that genome streamlining, a characteristic typical of obligate bacterial symbionts, is not prominent in the genus *Endozoicomonas* [13, 32, 34]. This is further supported by their high metabolic versatility along with the existence of a free-living stage, as indicated by low environmental abundance of *Endozoicomonas* in the water column surrounding corals [35].

Assessing the function of coral-associated bacteria is challenging because only a minuscule fraction of marine bacteria is cultivable [33, 36]. Further, sequencing approaches in holobionts may be confounded by an excess of host-derived reads compared to bacterial reads [37]. Moreover, while some species of *Endozoicomonas* have been successfully cultured from corals and other marine animals [14, 32], there are also reports of strains that are not readily amenable to cultivation [13, 38, 39]. Consequently, only a few *Endozoicomonas* genomes exist, but these indicate genomic capacity for rapid adaptation along with an ample metabolic diversity [9, 13, 14, 34, 40]. Less understood, however, is the role of *Endozoicomonas* in the coral holobiont and how the associated cues prime the bacterium for symbiosis.

The aim of this study was to identify potential interactions of *Endozoicomonas* with other members of the coral holobiont, and hence, their potential contribution to the health, acclimatization, and adaptation of the holobiont. To accomplish this, we cultured an *Endozoicomonas* isolate (strain 6c) from the common Red Sea coral *Acropora humilis*. The subsequent generation of (i) a high-quality draft genome of *Endozoicomonas* strain 6c in conjunction with (ii) transcriptomic and proteomic responses of the cultured isolate to tissue extracts of its coral host (i.e., holobiont cues) allowed us to identify putative interactions within the holobiont.

## Material and methods

### Tissue-associated bacterial community characterization of the coral *Acropora humilis*

For characterization of the bacterial community composition, finger-sized fragments of six colonies of *A*. *humilis* were collected on a shallow-water fringing reef close to the Saudi Arabian central Red Sea (Abu Shosha Reef; 22°18′16.3″N, 39°02′57.7″E). Care was taken to select corals >15 m apart to avoid clonal colonies, i.e., to increase the likelihood that different coral genotypes were collected. Corals were brought back to the lab in <1 h, snap-frozen in liquid nitrogen, and stored at −80 °C until further processing. For total RNA extraction, each fragment was doused in 1 ml of RLT buffer (Qiagen, Hilden, Germany) and tissue was removed from the skeleton by air-blasting using pressurized air through a 1000  µl barrier tip. Tissues were mechanically homogenized on ice using an UltraTurrax (T 18 basic, IKA Labortechnik, Staufen im Breisgau, Germany) at maximum speed for 15 s. Total RNA from the coral tissue homogenate, along with a negative RNA extraction (using only kit reagents to account for potential contamination), was extracted using 100 µl aliquots and the RNeasy Mini Kit (Qiagen, Hilden, Germany), following the manufacturer’s instructions. To remove genomic DNA, a DNase treatment was performed on the column following the manufacturer’s instructions. RNA quantity and integrity were assessed using a Qubit 2.0 fluorometer (Invitrogen, Waltham, US) and BioAnalyzer (Agilent Technologies, Santa Clara, US), respectively. Total RNA was used for cDNA synthesis by reverse transcription using the SuperScript First-Strand Synthesis System (Invitrogen, Waltham, United States), according to the manufacturer’s instructions. For amplification of the hypervariable regions v5 and v6 of the 16S rRNA gene for metabarcoding from cDNA, the primer pair 784F-1061 R [41, 42] with MiSeq overhang adapter sequences were used: forward: 5′- TCGTCGGCAGCGTCAGATGTGTATAAGAGACAGAGGATTAGATACCCTGGTA-3′; reverse: 5′-GTCTCGTGGGCTCGGAGATGTGTATAAGAGACAGCRRCACGAGCTGACGAC-3′; Illumina overhang adaptor sequences are underlined). Of note, this primer pair works well with marine samples, including corals (e.g., [31, 42, 43]. PCR reactions were performed in triplicate using the Qiagen Multiplex PCR kit (Qiagen, Hilden, Germany) with 1 µl of cDNA and a primer concentration of 0.5 μM in a reaction volume of 10 µl. Thermal conditions for the PCRs were as follows: initial denaturation at 95 °C for 15 min, 27 cycles of 95 °C for 30 s, 55 °C for 90 s, 72 °C for 30 s, followed by a final extension step at 72 °C for 10 min. In addition, a null template (no cDNA input) ‘negative’ control reaction was run to assess for PCR reagent contamination. Triplicate PCRs for each sample were pooled and cleaned with Illustra ExoProStar 1-Step (GE Healthcare, Chicago, US). Samples were subsequently indexed (dual indices and Illumina sequencing adapters attached in eight PCR cycles) using the Nextera XT Index Kit v2 (Illumina, San Diego, US). Indexed PCR products were normalized using the Invitrogen SequalPrep Normalization Plate Kit (Invitrogen, Waltham, US), pooled in equimolar ratios, and concentrated using a CentriVap Benchtop Vacuum Concentrator (Labconco, Kansas City, US). Pooled samples were quality checked on an Agilent 2100 BioAnalyzer (Agilent Technologies, Santa Clara, US) before sequencing. The library went through a further purification step using Agencourt AMPure beads (Agencourt Bioscience Corporation, Beverly, US). The library was sent for sequencing to Macrogen Korea with 2 × 250 bp on a HiSeq 2500 (Illumina) according to the manufacturer’s specifications.

### Isolation of *Endozoicomonas* from the coral *Acropora humilis* and absolute quantification of the isolate in coral tissues using qPCR

One finger-sized fragment of *A. humilis* was collected from Abu Shosha reef in June 2017 (at a depth of 5 m). The coral was maintained overnight in seawater from the collection site in flow-through aquaria (28 °C, salinity 40 PSU) and processed for bacterial isolation the following morning. In brief, coral tissue was removed from the skeleton with a clean air gun and autoclaved filtered seawater (AFSW; filter: Whatman, 0.22 µm). A total volume of tissue slurry of 15 ml was homogenized for 30 s at 3500 rpm with an UltraTurrax (T 18 basic, IKA, Staufen im Breisgau, Germany). Slurry was plated on Marine Agar 2216 (MA; BD Difco) following the standard dilution method (1:10, 1:100, and 1:1000 dilutions; for full details, refer to [44]. After incubation at 23 °C for 4 days, *Endozoicomonas* strain 6c was purified as a single colony by standard colony picking and quadrant-streaking technique onto a fresh MA plate (minimum three passages). Colonies are beige, convex, and with entire margins, and have a colony diameter of 2–3 mm on MA after 72 h incubation at 23 °C. Colonies are very sticky on marine agar and difficult to break up by vortexing in suspension. Cells are gram-negative motile rods (0.5–1.0 μm in diameter, 1.0–3.0 μm long). The strain was subsequently preserved at −80 °C as a 25% (v/v) glycerol suspension in marine broth 2216 (MB; BD Difco). For genotyping, colony PCR amplification was performed on the full-length of the 16S rRNA gene using the primers 27F 5′-AGAGTTTGATCCTGGCTCAG-3′ and 1492R 5′-GGTTACCTTGTTACGACTT-3′ with the following PCR conditions: 95 °C for 15 min, followed by 35 cycles of each: 30 s at 95 °C, 90 s at 55 °C, and 90 s at 72 °C [45]. A final extension step was set at 72 °C for 10 min. Post-PCR cleanup was performed by adding 2 µl of Illustra ExoProStar 1-Step to 10 µl of PCR product and following the manufacturer's instructions (GE Healthcare Life Sciences, Solingen, Germany). Cleanedup PCR products were sent to the KAUST Bioscience Core Lab for Sanger sequencing; the full-length 16S rRNA gene sequence confirmed the isolated strain was affiliated to the genus *Endozoicomonas*.

The full-length 16S rRNA gene sequence obtained from Sanger sequencing was used to design a specific primer pair for *Endozoicomonas* 6c. Full-length 16S rRNA gene sequences of *Endozoicomonas* 6c and that of other *Endozoicomonas* for which genomes are available were aligned using the alignment editor in MEGA 7 [46]. The resulting taxon-specific primer Endoz-6c-F and Endoz-6c-R (forward: 5′-TCGTCGGGGATCTTGCATTT-3′; reverse: 5′-AGGATTCGCAGGATGTCAAGG-3′) amplifies a 180 bp long region of the 16S rRNA gene of *Endozoicomonas* 6c. Running the primer sequences through the SILVA TestPrime tool [47] revealed only one match from a partial sequence from a 16S rRNA gene amplicon sequencing data set from the Red Sea coral *Stylophora pistillata* (accession number KC668564; [42]). Primers were checked on a 1% agarose gel for single bands after PCR amplification using the following protocol: 95 °C for 15 min, followed by 35 cycles of 95 °C for 30 s, 55 °C for 40 s, and 72 °C for 30 s, and a final extension step of 72 °C for 10 min.

For absolute quantification of 16S rRNA gene copy numbers of *Endozoicomonas* 6c in the tissues of its native coral host, total RNA from the same samples from which 16S rRNA gene sequencing data were generated was used. Lyophilized total RNA of *Acropora humilis* (*n* = 6) was reconstituted from GenTegra RNA plates (NBS Scientific, Canonsburg, USA) following the manufacturer’s instructions and quantified using Qubit (Qubit RNA High Sensitivity Assay Kit, Invitrogen). Subsequently, 200 ng of total RNA were aliquoted from each sample for DNase treatment (Qiagen, Hilden, Germany) to remove any residuals of genomic DNA and then used as input for single-stranded cDNA synthesis using the High Capacity cDNA Reverse Transcription Kit (Applied Biosystems, Waltham, US). For absolute quantification using a quantitative PCR (qPCR) approach, standard curves were first generated from PCR products from one *A*. *humilis* sample using the described 16S rRNA gene universal bacterial primers and the *Endozoicomonas* 6c-specific primers. Following electrophoresis on a 0.8% agar gel, amplicon gel slices from different samples were cut out, and the DNA was purified using the QIAquick gel extraction kit (Qiagen, Hilden, Germany) following the manufacturer’s instructions and quantified using a Qubit fluorometer. All qPCR reactions (cDNA from *A*. *humilis* total RNA samples plus standards for the calibration curve) were run on a qTOWER^3^ 84 using the innuMIX qPCR DSGreen Standard master mix (both Analytik Jena GmbH, Germany), with 0.2 μl each of 10 μM forward and reverse primers to target the entire bacterial community and *Endozoicomonas* 6c to target the proportion of this strain in each sample, respectively. The qPCRs were run in reaction volumes of 10 μl using the following thermal profile: 95 °C for 2 min, 50 cycles of 95 °C for 30 s, 55 °C for 40 s, 72 °C for 30 s, and a subsequent melting curve analysis to assess uniformity of amplification and to confirm the absence of primer dimers. All reactions were run in technical triplicates in addition to a no-template control for both primer pairs. Absolute quantification of 16S rRNA and *Endozoicomonas* 6c gene copy numbers was performed by interpolating qPCR Ct values against the standard calibration curve of known gene copies. Subsequently, the proportion of *Endozoicomonas* 6c in the total bacterial community was calculated from absolute gene copy numbers of both and expressed as mean percentage for the *A*. *humilis* samples.

### Genome sequencing and assembly

*Endozoicomonas* 6c was grown in Marine Broth (BD Difco 2216) under constant agitation (60 rpm) at 25 °C until OD_600_ = 0.4 and harvested after 48 h in mid-exponential phase. High-molecular weight genomic DNA (HMW gDNA) was extracted using the Genomic-Tip 100/G kit (Qiagen, Hilden, Germany) following the manufacturer’s instructions for gram-negative bacteria. Quality control and library preparation for long-read sequencing on the PacBio RSII platform was conducted at the KAUST Bioscience Core Lab. In brief, concentration of HMW gDNA was assessed on a Qubit fluorometer. Sufficient quality of HMW gDNA for PacBio sequencing (260/280 of 1.8–2, 260/230 >2) was confirmed on a NanoDrop 2000C spectrophotometer (Thermo Fisher Scientific, Waltham, US). Fragment size distribution was assessed on a fragment analyzer (Agilent Biosystems, Santa Clara, US); the average fragment size of the DNA was 23,711 bp. Finally, genomic DNA library preparation was performed following PacBio’s procedure & checklist for a 20-kb template preparation using the BluePippin Size-Selection System with a library insert size of 10 kb. The resulting library was sequenced on one flow cell on the PacBio RSII platform.

Genome assembly was performed using canu v1.6 [48] with the option “-pacbio genomeSize = 5.0m” and error correction mode. The assembled genomic contigs (*n* = 3) were checked for completeness (97.02%) and contamination (0.54%) using CheckM v1.1.0 [49] with the lineage-specific option. The assembled genome was annotated using RAST [50]. For characterization of genomic features of *Endozoicomonas* 6c putatively relevant to host-microbe interactions, protein families (Pfams) were predicted using the online server WebMGA [51] using the amino acid fasta file from RAST.

### Phylogenetic placement

For phylogenomic inference and tree-building, publicly available *Endozoicomonas* genomes were obtained from NCBI and RAST (accession date: January 2018). Genomes obtained from NCBI included (assembly numbers and original reference in parentheses): *E*. *acroporae* Acr-14 (GCA_002864045.1; [52]); *E. arenosclerae* ab112 (GCA_001562015; [53]); *E*. *numazuensis* DSM 25634 (GCA_000722635; [40]); *E*. *montiporae* CL-33 (GCA_001583435; [40]); *E*. *elysicola* DSM 22380 (GCA_000373945; [40]); *E*. *atrinae* WP70 (GCA_001647025; [54]); *E*. *ascidiicola* AVMART05 (GCA_001646945; [55]); *Endozoicomonas* sp. AB1_5 (GCA_001729985; [56]). Genomes of coral-associated *Endozoicomonas* obtained from RAST included (RAST IDs in parentheses; all obtained from [13]): *Endozoicomonas* from *Stylophora pistillata*, henceforth *E.* ‘pistillata’ type A (6666666.127878) and *E*. ‘pistillata’ type B (6666666.127879); from *A*. *humilis*, henceforth *E*. ‘humilis’ (305899.13); and from *Pocillopora* *verrucosa*, henceforth *E*. ‘verrucosa’ (305899.6). For species delineation, Genome-to-Genome Distance Calculation (GGDC) [57] was performed using the online server of the German Collection for Microorganisms and Cell Cultures (http://ggdc.dsmz.de). Amino acid identities and average nucleotide identities were performed using the online ANI/AAI calculator tool of the enveomics collection [58]. Phylogenomic inference was performed through the OrthoFinder2 default workflow following ortholog prediction on amino acid fasta files of *Endozoicomonas* genomes using OrthoFinder2 v2.5.4 [59]. OrthoFinder2 was used to infer conserved orthologs among the genomes, and followed by Multiple Sequence Alignment (MSA) using MUSCLE [60]. The MSA was then used to construct a consensus tree based on the topology of trees for all genes as described in detail elsewhere [59]. Finally, the unrooted species tree was visualized in FigTree v1.1.4 [61]. In addition, we performed a comparative approach to screen for the presence of protein domains associated with DMSP catabolism by annotating the genomes of *Endozoicomonas* 6c and that of the other *Endozoicomonas* using Prokka v1.13 [62], KOfamScan v1.3.0 [63] against KEGG [64], and MMseqs2 v11.e1a1c against UniProt (downloaded on 04-21-20) [65].

### Cell culture experiment

#### Preparation of coral host tissue extract

Coral host tissue extracts were prepared following [66, 67]. Five colonies of *A. humilis* were collected at Abu Shosha Reef in January 2018. Corals were transported back to the lab within an hour of collection and maintained at 28 °C for 48 h in a 12:12 h light-dark regime resembling natural conditions (mean daytime radiation 380 µmol quanta m^−2^ s^−1^, peak daytime irradiance 750 µmol quanta m^−2^ s^−1^; Radion light system, Ecotech Marine Inc.). Coral fragments were then doused in 2 ml AFSW collected from Abu Shosha Reef, followed by tissue removal through air-blasting. Resultant coral slurry was homogenized using an UltraTurrax (30 s, 3500 × rpm; T 18 basic, IKA Labortechnik, Staufen im Breisgau, Germany) and centrifuged at 4 °C and 3000 *g* for 3 min to pellet algal symbiont cells. The crude homogenate, i.e., algal symbiont-free, cell-free host supernatant was decanted, transferred to Amicon-15 3K centrifugal filter units (Merck, Kenilworth, USA), and fractionated to 3 kDA by centrifugation at 4 °C and 3000 × *g* for 80 min. Ultra-fractionated coral host tissue extracts originating from different fragments of *A*. *humilis* were pooled, snap-frozen, and subsequently stored at −20 °C for less than 14 days, until the cell culture experiment [67].

#### Cell culture conditions and incubations

**Fig. 1:**
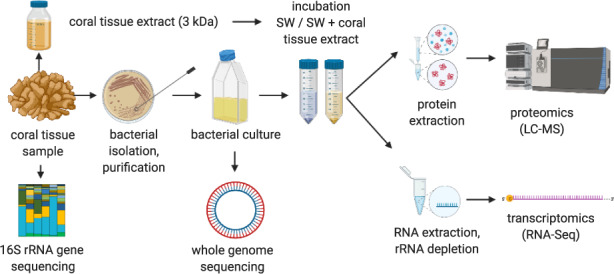
Overview of experimental approach. *Endozoicomonas* 6c was isolated from the tissues of the Red Sea coral *Acropora humilis*. From the bacterial culture, we generated (i) a high-quality draft genome and (ii) a metabolic reconstruction based on transcriptomic and proteomic responses of *Endozoicomonas* 6c to tissue extracts of its native coral host in vitro.

A cell culture-based experiment to investigate the response of *Endozoicomonas* 6c to two experimental conditions (i.e., control and host tissue ultrafiltrate, from now on referred to as ‘extract’) for subsequent transcriptomic and proteomic analyses was conducted (for a schematic summary of the experimental approach, please refer to Fig. 1). Prior to manipulation experiments, growth curves of *Endozoicomonas* 6c in AFSW and AFSW + 15% host tissue extract were assessed. For this, 200 μl sterile aliquots of AFSW, AFSW + 15% host tissue extract, and Difco2216 Marine Broth were transferred into a clean, clear flat-bottom 96-well plate in two sets of triplicate wells each. For each of the three media, one set of triplicate wells was inoculated with 2 μl of bacterial culture (at a density of ~10^5^ cells ml^−1^), the inoculated Marine Broth serving as a positive control for growth. The second set of triplicate wells was not inoculated and served as a ‘blank’ for plate reader measurements of the respective medium. Optical density (OD_600_) measurements were performed in a plate reader (SpectraMax Paradigm, Molecular Devices LLC, San José, USA) immediately after inoculation (0 h) as well as after 24 and 48 h of incubation under constant agitation (60 rpm) at 28 °C. Of note, no growth was observed in AFSW and AFSW + 15% host tissue extract (Supplementary Fig. S1). While this could reflect potential effects of nutrient starvation on the *Endozoicomonas* 6c cells, we were at the same time able to rule out any confounding effects due to differential growth in the two experimental conditions. For the experiment, *Endozoicomonas* 6c cells were grown overnight at 28 °C in a batch culture (500 ml) under constant agitation (150 rpm). Inoculation was realized with cells from pre-cultures in mid-exponential phase (2.3 × 10^5^ cells ml^−1^) grown in 2216 Difco Marine Broth. Replicate aliquots of 50 ml (2.6 × 105 cells ml^−1^) were centrifuged at 3000 × *g* for 10 min in a swing-bucket centrifuge. The supernatants were discarded and cell pellets resuspended in AFSW. Pelleted cells used for the control condition were resuspended in 50 ml AFSW. Pelleted cells intended for incubation in ultra-fractionated host tissue extract were resuspended in 42.5 ml ASW + 7.5 ml of host tissue extract (final proportion 15%). Cells in both treatments were aliquoted (*n* = 6 aliquots for each treatment and for transcriptomic and proteomic analyses each; Fig. 1; 8 ml aliquot volume at a density of ~2.6 × 10^5^ cells ml^−1^) and incubated in 15 ml Falcon tubes under constant agitation at 28 °C for 3 h. Cells for transcriptomic analysis were pelleted at 3000 × *g* at room temperature for 10 min. Pelleted cells were washed once in 2 ml 2 × PBS, pelleted again, resuspended in 2 ml RLT buffer (Qiagen, Hilden, Germany) in sterile nuclease-free Eppendorf tubes, immediately snap-frozen in liquid nitrogen, and stored at −80 °C. Cells for proteomic analyses were spun down at 4000 × *g* for 10 min, washed once in 2 ml 2 × PBS, pelleted again, immediately snap-frozen in liquid nitrogen, and freeze-dried for 24 h. Snap-frozen cells for transcriptomic analysis were processed at KAUST (SA), freeze-dried cells for proteomic analysis were shipped to Victoria University of Wellington (NZ) for protein extraction and LC-MS analysis (see below for details on sample processing) (for a schematic summary of the experimental approach, please refer to Fig. 1).

To assess cell numbers in the pre-culture, experimental culture, and from each of the two treatments at the beginning and the end of the incubation, 1 ml aliquots were set aside for enumeration with flow cytometry. In each aliquot, cells were pelleted at 3000 × *g* for 15 min and the pellet resuspended in 2 × PBS. Cells were pelleted again and rapidly resuspended in 2 × PBS containing 4% paraformaldehyde. Cells were fixed at 4 °C for 4 h. After fixation, cells were pelleted at 3000 × *g* for 15 min and resuspended in 2 × PBS. Cells were subsequently stained with DAPI (working concentration 5 µg ml^−1^, staining for 15 min in the dark at RT) and analyzed by flow cytometry in the presence of the DAPI dye (405 nm/488 nm excitation/emission, BD LSR Fortessa, BD Biosciences, Franklin Lakes, US). Gating of recorded events was performed in FlowJo v.10.5.3. based on forward scatter and DAPI fluorescence. Aliquots collected at the beginning of the incubation period contained an average of 2.7 × 10^5^ cells ml^−1^, and 2.6 × 10^5^ cells ml^−1^ after the incubation period.

### RNA extraction, rRNA depletion, and RNA-Seq library preparation

To obtain bacterial mRNA, snap-frozen homogenized *Endozoicomonas* 6c cells in RLT buffer from the cell culture experiment were thawed on ice. 200 µl aliquots in an additional 350 µl RLT buffer were used for total RNA extraction using the RNeasy Mini Kit (Qiagen, Hilden, Germany) following the manufacturer’s instructions. To remove genomic DNA, a DNase treatment was performed following the manufacturer’s instructions. Purified total RNA was quantified using a Qubit fluorometer using the high-sensitivity RNA kit (Invitrogen, Waltham, US). For some samples, it was necessary to perform and pool multiple total RNA extractions from the same sample aliquot, and to pool total RNA (previously precipitated with 1/10th volume 3 M sodium acetate pH 5.2 and glycogen at 5 mg ml^−1^ final concentration). Large ribosomal RNAs (16S rRNA, 23S rRNA) were depleted using the Ambion MICROBExpress kit (Life Technologies, Carlsbad, US). Depletion of large rRNAs was confirmed on an Agilent 2100 BioAnalyzer (Agilent, Santa Clara, US). Samples were then subsequently depleted of small RNAs (5S rRNA, tRNA) using the MEGAclear TranscriptionClean-Up kit (Invitrogen, Waltham, US). The remaining enriched bacterial mRNA (input normalized to up to 100 ng) was used for library preparation using the TruSeq Stranded Total RNA Library Prep kit (Illumina) according to manufacturer instructions. The resulting libraries (average fragment size of 314 bp) were sequenced on a HiSeq 4000 platform (Illumina) at the Bioscience Core Lab facilities at KAUST to obtain paired-end reads of 2 × 150 bp.

### Protein extraction, digestion, and peptide purification

Protein extraction and separation were based on the filter-aided sample preparation methods of [68]. The cell pellet was resuspended and the proteins solubilized by ultrasonicator probe, 20 × 2 s pulses, in 5% sodium deoxycholate. The dissolved proteins were denatured at 85 °C for 30 min with 1% final concentration β-mercaptoethanol. Lipids and detergent were reduced by washing the aqueous protein sample twice with two volumes of ethyl acetate, followed by phase separation and removal of the upper organic phase. Any remaining ethyl acetate was eliminated by 20 min vacuum centrifugation. Samples were concentrated in a 0.5 ml Amicon Ultra 30 kDA centrifugation filter (14,000 × *g*, Merck Millipore, Burlingham, US) followed by two washes with 380 µl 50 mM Tris buffer, pH 8.1 followed by resuspension in 400 µl total Tris buffer. The protein content of a subsample, acidified and centrifuged (22,000 × *g*, 5 min) to remove remaining deoxycholate, was quantified by a Qubit fluorometer. 10 mM β-mercaptoethanol was added to 100 µg total protein in the centrifugation filter and incubated for 10 min at 37 °C, followed by alkylation with 20 mM acrylamide for 20 min at room temperature and quenching with a second addition of β-mercaptoethanol. Proteins were then digested with 2 µg trypsin for 18 h and the digested peptides separated by filter centrifugation. Any remaining deoxycholate was precipitated by adding formic acid (1% final) and centrifugation (16,000 × *g*, 1 min). Peptides were desalted by C18 pipette tips (Omix Bond Elut, Agilent Technologies, Santa Clara, US), dried by vacuum centrifugation, and stored at 4 °C. For analysis, peptides were dissolved in 50 µl 0.1% formic acid and quantified by Qubit fluorometry.

### Liquid chromatography-tandem mass spectrometry

A 75 min linear gradient (5–35% buffer B) was used to separate peptides at 300 nl min^−1^ (buffer A: 0.1% formic acid; buffer B: 80% acetonitrile, 0.1% formic acid) with a 15 cm column (Acclaim PepMap C18, 100 Å, 3 µm, Thermo Scientific, Auckland, New Zealand) on an Ultimate 3000 liquid chromatography system (Dionex, Sunnyvale, US). An Orbitrap Fusion Lumos Tribrid mass spectrometer was used to analyze peptides by electrospray ionization (1.8 kV). Each sample was analyzed twice. The Orbitrap acquired precursor mass spectra with a resolution of 120,000 while rejecting singly-charged ions, with an automatic gain target of 7.0e5, maximum injection time 50 ms, and quadrupole isolation enabled. High-energy collision dissociation was used for fragmentation and the twenty most intense precursor spectra were analyzed by ion trap (maximum injection time 300 ms, automatic gain target 5.0e3) and dynamic exclusion (60 s) enabled.

### Protein identification, quantification, and data analysis

Protein identification was performed using MaxQuant (1.6.10.43, [69, 70]), with the raw spectra searched against *Endozoicomonas* 6c protein models generated as below. A minimum of two peptides was required to be considered a valid match, and peptide and protein search false discovery rates had a maximum of 1%. N-terminus acetylation and methionine oxidation were valid variable modifications and carbamidomethylation a valid fixed modification, with a maximum of two missed tryptic cleavages. Peptide search tolerances for the first and main searches were 20 and 4.5 ppm, respectively, with a mass tolerance of 0.5 Da in the ion trap. Label-free quantification and match between runs were enabled, with a quantification minimum of two unique plus razor peptides.

### Data analysis and statistics

#### Analysis of 16S rRNA gene sequencing data

Demultiplexed raw sequence reads were processed using the DADA2 workflow for exact amplicon sequence variants (ASVs) for 16S rRNA sequencing data. The resulting sequences were then processed using DADA2 [71]. The error model was built and inspected using the ‘learnErrors’ and ‘plotErrors’ commands as implemented in DADA2. Denoised reads were then merged (265,837 merged read pairs retained) and chimeric contigs discarded using ‘mergePairs’ and ‘removeBimeraDenovo’, respectively; after chimera removal, 233,375 merged sequences were retained and considered ASVs. ASVs with incidence <10 cumulatively over all samples were discarded from further analyses. Finally, ASVs found in sequenced ‘negative’ DNA extraction comprising more than 5% of sequences from *A*. *humilis* samples were considered contamination and discarded (ASV510, *Bosea*; ASV51 and 564, *Pelomonas*; ASV116, Rhizobiales), leaving a total of 169,672 sequences with an average length of 283 bp distributed over 480 unique ASVs (averaging ~29,000 sequences per sample). Taxonomic ranks were assigned based on the SILVA database version 138 [72], using DADA2 function ‘assignTaxonomy’. All raw sequence data are accessible under NCBI BioProject PRJNA753662.

#### Analysis of transcriptomic and proteomic data

RNA sequence reads (samples: control *n* = 3, host tissue extract treatment *n* = 5) were quality trimmed, Illumina adapters were removed, and short reads with low-quality scores discarded using Trimmomatic v.0.39 [73]. The successful removal of adapters from paired reads was confirmed using FastQC v.0.11.5 [74]. Paired reads were mapped to the gene models of the assembled *Endozoicomonas* 6c genome using BBmap (BBtools v.37.10) [75] to generate BAM files, which were then used as input in Salmon v.1.0.0. [76] to quantify gene expression using the alignment-based mode. Effective counts were used for identifying significantly differentially expressed genes (FDR-adjusted *p*  < 0.05) between pairs of treatments using DESeq2 v.1.26.0 [77]. Genes were assigned to GO and KEGG categories using eggNOG 4.5.1. [78]. Variance stabilizing transformation was applied to count data for principal component analysis and visualization of similarity between transcriptome-wide expression profiles as implemented in DESeq2.

The protein data (samples: control = 6, host tissue extract treatment *n* = 5) were pre-processed in Perseus (1.6.10.45, [79]), removing contaminant proteins, decoy sequence matches, and proteins only identified by site, and log2-transforming the protein label-free quantification intensities. PolySTest [80] was used to determine proteins that were significantly differentially abundant between treatments by false discovery rate (FDR) using the limma algorithm (FDR < 0.05, fold change threshold: |FC| > 0.5). The mass spectra are available via the PRIDE partner repository [81] with the dataset identifier PXD027178 and DOI 10.6019/PXD027178.

Differentially expressed transcripts and proteomic features were used to perform enrichment analyses with topGO v.2.38.1 using the ‘weight01’ algorithm and no multiple test correction, as recommended [82]. Transcriptomic and proteomic responses were assessed separately due to known methodological biases. To assess consistently regulated features present in both datasets, lists of overlapping features between transcriptome and proteomic datasets and their directions of change were generated. The list was then run through topGO v.2.38.1 as described above, and affiliated biological processes (GO terms) statistically tested using Fisher’s exact test. Pathways of interest were further investigated by mapping differentially expressed genes to KEGG pathways using KEGG Mapper v.3.2 [64]. For the visualization of direction and significance of expression change of significant GO terms, *z*-scores and negative logarithms of the adjusted *p* values were computed for lists of significant GO terms associated with the experimental treatment for both transcriptomic and proteomic datasets and used as input to generate bubble plots using the R package GOplot [83]. For functions of interest identified in the transcriptomic and proteomic responses, we compared selected gene families across *Endozoicomonas* genomes and clades. Ortholog prediction was performed on amino acid fasta files of *Endozoicomonas* genomes using OrthoFinder2 v2.5.4. [59] with default settings. The resulting gene cluster matrices were then annotated in eggNOG-mapper v2 [78] and Pfam 24.0 [51] using the respective online platforms. Copy numbers of the considered gene families were then normalized to the size of each respective genome, resulting in a common metric of gene copies *per* megabase). An unrooted species tree of the 13 *Endozoicomonas* genomes used was also generated through the OrthoFinder2 default workflow, which was visualized using FigTree v1.1.4 [61]. Figures summarizing selected features of reconstructed transcriptomic and proteomic responses were created using BioRender.com.

## Results and discussion

### Bacterial community characterization of the Red Sea coral *A. humilis*

The bacterial community associated with *A*. *humilis* was dominated by Gammaproteobacteria, Bacteroidetes, and Alphaproteobacteria (58.1%, 15.5%, and 14.8% average relative abundance; Supplementary Fig. S2A). Sequences annotated to *Endozoicomonas* averaged 65.3% and 37.9% of Gammaproteobacteria and the total bacterial community, respectively (Supplementary Fig. S2B). Of 480 exact ASVs identified, 30 were annotated to *Endozoicomonas*, including the most abundant ASV 1 (Supplementary Table S1).

Querying all *A*. *humilis*-associated *Endozoicomonas* ASVs against the full-length 16S rRNA gene sequence of the isolated *Endozoicomonas* 6c using the BLASTN tool on NCBI, it matched ASVs 27, 35, 42, 43, 130 (>97 sequence % similarity). Together, these ASVs comprised about 2.8% of all sequences, suggesting that the novel isolate occurs at comparatively low abundance. This low relative abundance of *Endozoicomonas* 6c in the tissue-associated bacterial community of its native holobiont is supported by absolute quantification using qPCR, which suggests that the isolate occurs at a relative abundance of around 1.1% (±0.5% SE; Supplementary Table S2) of the total 16S rRNA gene copy numbers.

### The genome of *Endozoicomonas* 6c

**Fig. 2:**
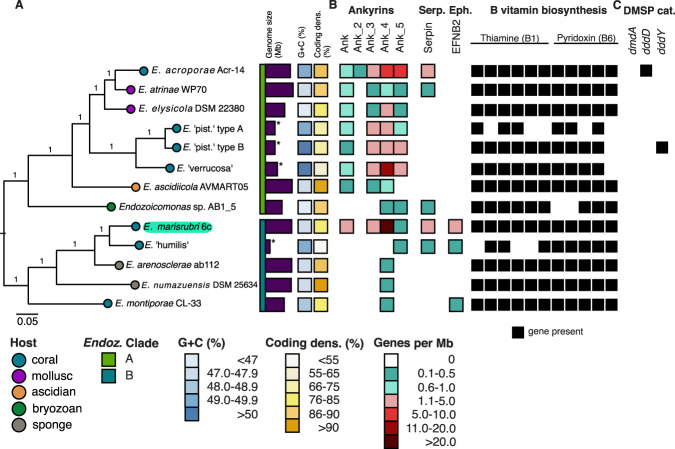
Phylogenetic relationship and genomic characterization of *Endozoicomonas *genomes. **A** Phylogenetic placement of the novel *Endozoicomonas marisrubri* 6c isolate (highlighted in turquoise) from the Red Sea coral *Acropora humilis*. The unrooted species tree was generated through the OrthoFinder2 default workflow and visualized in FigTree v1.1.4 [61]. Vertical bars show the two clades of *Endozoicomonas* and respective genome sizes (purple; asterisks refer to genomes of ≤90% completeness), boxes indicate G+C content (in %; blue hues), and coding density (in %; yellow hues) of the respective genomes. **B** Distribution of genes and functions proposed to be involved in symbiotic establishment and maintenance across *Endozoicomonas* clades and genomes according to their associated gene families (ortholog clusters) and Pfam profiles. The color code used for ankyrins, serpins, and ephrins from turquoise to red reflects the number of genes *per* million base pairs (Mbp) of genome. Black boxes for genes associated with B vitamin biosynthetic gene clusters reflect their presence within the respective genome. **C** Distribution of protein domains associated with DMSP catabolism across *Endozoicomonas* genomes based on annotation with Prokka, KEGG, and UniProt. Black boxes reflect the presence of protein domains within the respective genome.

The assembled draft genome of *Endozoicomonas* 6c of 7.69 Mb was estimated to be 97.02% complete, with 7226 predicted coding sequences (CDS), a coding density of 83%, and a G+C content of 47.8% (Fig. 2A). Contamination was low, as estimated by CheckM, at 0.54%. The genome was assembled into three contigs with an N50 of 4,568,499 bp. Based on the above, in addition to the presence of tRNAs for all 20 proteinogenic amino acids, this genome can be classified as a ‘high-quality draft’ [84]. The genome harbors seven copies of the 16S rRNA gene, which are organized in six operons. Of note, long-read sequencing technologies as employed in this study can be prone to systematic high error rates. However, the characterized genome was sequenced at high coverage (>250×), is nearly complete, with a large number of genes and high coding density in line with that of other *Endozoicomonas* genomes, and thus, fulfills all criteria to be classified as a high-quality draft.

Of the 3605 genes in the genome of the novel *Endozoicomonas* 6c assigned to SEED-annotated subsystems as implemented by RAST, 11.2% encode for cellular structural components and processes; 13.8% for nucleotide, nucleoside, and nucleic acid metabolism; 6.0% for regulation, cell signaling, chemotaxis, and motility; 8.1% for cofactors, vitamins, prosthetic groups, and pigments; 11.2% for metabolism and elemental cycling; and 7.1% for stress responses, virulence, disease, and defense (Supplementary Table S3). Similar to what has been reported for other Red Sea *Acropora*-associated *Endozoicomonas* genomes, the genome of *Endozoicomonas* 6c contains high numbers of putative protein families previously suggested to be relevant for host infection and symbiosis establishment and maintenance [33, 85, 86], such as ankyrin repeats (784 CDSs, or 102 genes *per* Mbp), WD40 repeats (1555 CDSs, or 202 genes *per* Mbp), and tetratricopeptide repeats (300 CDSs, or 39 genes *per* Mbp). Bacterial secretion systems previously implicated in host-microbe or microbe-microbe interactions were identified (44 CDSs or 6 genes *per* Mbp affiliated to type II secretion system T2SS, 157 CDSs or 20 genes *per* Mbp affiliated to type III T3SS, 12 CDSs or ~2 genes *per* Mbp affiliated to type IV T4SS, and none to type VI secretion system T6SS). Of note, the genome of *Endozoicomonas* 6c harbors a greater number of CDSs affiliated to T3SS, but fewer CDSs affiliated to T2SS and T4SS compared to other coral-associated *Endozoicomonas* [33]. In addition, a minimum of 449 CDSs (58 genes *per* Mbp) pertaining to mobile elements (eight group II introns or 1 *per* Mbp; 23 integrases or 3 *per* Mbp, and 422 transposases or 55 *per* Mbp) were identified by the Pfam query. Finally, the genome of *Endozoicomonas* 6c contains a full type 1 CRISPR array (*csy* proteins 1 to 4) as well as the CRISPR-Cas3 helicase.

From a metabolic point of view, the genome encodes for biosynthetic gene clusters for multiple vitamins, cofactors, and amino acids. Notably, these include the B vitamins thiamine (B1), riboflavin (B2), pyridoxine (B6), biotin (B7), and folate (B9), which are essential for animals and many algae. The presence of genes encoding for the cofactors flavodoxin, lipoic acid (lipoate), coenzyme A, NAD/NADP, quinones, heme, and siroheme was also confirmed. Overall, more than 500 genes associated with the metabolism of amino acids and their derivatives were annotated in the *Endozoicomonas* 6c genome. These included, but were not limited to, the biosynthetic subsystems for arginine, the urea cycle, polyamines (137 genes), lysine, threonine, methionine, and cysteine (120 genes), branched-chain amino acids (72 genes), and aromatic amino acids and derivatives (59 genes). Some differences to other *Endozoicomonas* genomes are apparent with regard to the numbers of genes in individual (SEED) amino acid subsystems [13]. Overall, the numbers of annotated genes for amino acid metabolism in the genome of *Endozoicomonas* 6c are well within the expected range, although higher than in other *Endozoicomonas* genomes for individual subsystems (e.g., for the subsystems ‘arginine, urea cycle, polyamines’, and ‘lysine, threonine, methionine, cysteine’). Finally, no gene clusters associated with the metabolism of the osmolyte and antioxidant dimethylsulfoniopropionate (DMSP) were identified in the genome of *Endozoicomonas* 6c using the SEED-annotated subsystems approach, which contrasts with previous reports on the occurrence of genes for DMSP metabolism in *E. acroporae* from Taiwan [32].

The genome of *Endozoicomonas* 6c harbors protein families previously implicated in symbiosis establishment (ankyrin, WD40 and tetratricopeptide repeats, mobile elements; [33, 85, 86]). However, *Endozoicomonas* 6c has a large genome size, high metabolic diversity, and is culturable. Together with the existence of free-living stages of bacteria in the genus *Endozoicomonas* [35], this suggests that no genome streamlining has occurred [32], and that *Endozoicomonas* 6c is not an obligate, fully host-restricted coral-bacterial symbiont.

### Phylogenetic placement within the genus *Endozoicomonas*

Phylogenetic inference based on GGDC and ortholog prediction, as well as ANI and AAI [87] suggest that the new isolate may be highly similar to *E*. ‘humilis’, an uncultured *Endozoicomonas* associated with the Red Sea coral *A*. *humilis* and previously characterized by metagenomic binning [13] (dDDH of 35.1%, ANI and AAI values of 85 and 83, respectively; bear in mind the low completeness of the *E*. ‘humilis’ genome, which may affect these metrics). Together, the results of GGDC (in the range of 21.7–35.1%), percentage G+C differences (0.01–5.98%), phylogenetic placement, and ANI and AAI values (well below 95% and 90%, respectively) place strain 6c as a distinct species, for which we propose the name *E. marisrubri* (‘of the Red Sea’) sp. nov. 6c (Supplementary Tables S4, S5a, b). In the phylogenomic tree, *E. marisrubi* 6c (together with *E*. humilis) is placed closest to the two sponge-associated strains, *E*. *arenosclerae* and *E*. *numazuensis*, which together position as sister to *E. montiporae*, a coral-associated strain (100% bootstrap support) (Fig. 2A).

The novel *E. marisrubri* 6c appears to be less similar to *E*. *acroporae*, an *Endozoicomonas* isolated from an unknown species of *Acropora* collected from the coast of southern Taiwan [52], and is placed in a separate clade of *Endozoicomonas* by phylogenomic analysis (Fig. 2A). This observation suggests complex patterns of host-symbiont species co-diversification, geographical adaptation (i.e., *Acropora* hosts might harbor geographically distinct *Endozoicomonas*, as previously proposed for the coral genus *Stylophora* [9]), and/or could reflect environmental acquisition of *Endozoicomonas*, as suggested previously [9, 13].

## Responses of *Endozoicomonas* following exposure to coral host tissue extract

### Benefits and limitations of in vitro cell-host tissue extract assays

Deciphering the function of coral-associated bacteria is challenging for several reasons. First, there are well-known limits to bacterial cultivation, as only a minuscule fraction of bacteria are currently cultivable [32, 33]. While a few *Endozoicomonas* cultures are available, there are reports of strains not being readily amenable to isolation from host tissues [26, 38, 39]; for the present study: unsuccessful isolation of *Endozoicomonas* from Red Sea *Pocillopora verrucosa* and *Stylophora pistillata*; data not shown). Second, sequencing approaches to assess bacterial metabolism and activity in complex holobionts such as corals remain challenging due to high proportions of host nucleic acids that disproportionately skew sequencing coverage of microbiomes in ‘-omics’ datasets [37, 88, 89]. Under these considerations, the present study pursued a symbiont-centric in vitro approach to characterize the transcriptomic and proteomic responses of *E. marisrubri* 6c to coral host tissue extract. While this approach has its own limitations, such as the dependence on cultivability, and the artificial homogenization of the host “micro-environment” which may not reflect natural nutrient availability in the intact symbiosis (as likely reflected by the absence of growth in the presence of host tissue extract; Supplementary Fig. S1), it allows us to elucidate possible behavioral and metabolic responses of *E. marisrubri* 6c upon encountering its coral host environment and enables the identification of putative host-microbe interactions.

### Coral host tissue extract elicits transcriptomic and proteomic responses in *Endozoicomonas*

We found distinct transcriptomic and proteomic responses of *E. marisrubri* 6c to coral host tissue extracts (Supplementary Fig. S3A, B). Overall, there was no significant correlation between the overlapping differentially expressed/abundant transcripts and proteins of *E. marisrubri* 6c cells exposed to host tissue extract (Pearson correlation, *t* = −1.126, *df* = 1793, *r* = −0.027, *p* value = 0.2603) (Supplementary Fig. S3c). Such disparity is commonly observed and may reflect the different timescales of transcriptomic and proteomic adjustments [90, 91], as well as known methodological biases (e.g., underrepresentation of the membrane proteome; [92]). Consequently, we decided to analyze the two datasets separately to obtain a comprehensive view of the responses of *E. marisrubri* 6c to coral holobiont cues to identify putative molecular responses in the onset of coral-bacterial symbiosis.

**Fig. 3:**
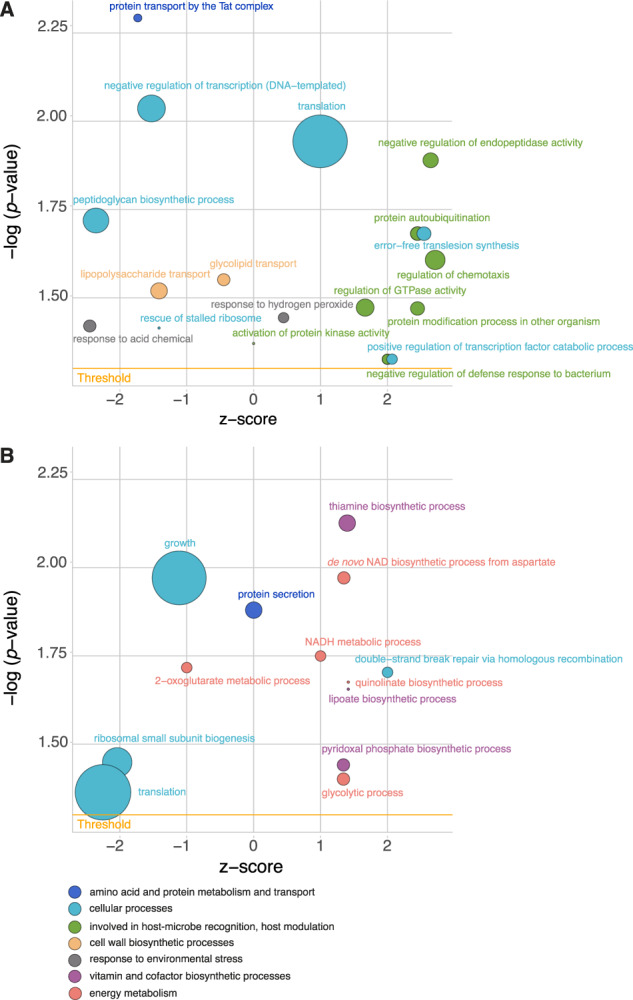
Direction of regulation in gene expression or protein abundance of significant biological processes (GO terms) associated with the response of *Endozoicomonas marisrubri* 6c to tissue extracts of its coral host (3 h exposure). **A** Transcriptomic response, **B** Proteomic response. Bubble size reflects the number of features (genes and proteins, respectively) within the respective GO term, color reflects overarching processes (parent terms). *Z*-scores (*x*-axis) reflect the overall direction of change of features within GO terms (*z* < 0: downregulation, *z* > 0 : upregulation). Threshold represents statistical significance (*p* < 0.05).

The sequenced transcriptome contained ~60 million read pairs that mapped to the genome of *E. marisrubri* 6c, distributed over control (*n* = 3) and host tissue-treated (*n* = 6) samples. Individual samples averaged around ~6.7 million read pairs. DESeq2 identified 633 differentially expressed genes (DEGs; 8.8% of the genome) between the control and cells exposed to host tissue extract. Of these, 285 were downregulated and 348 were upregulated, respectively, in the host tissue treatment (3.9 and 4.8% of the genome, respectively) after 3 h of exposure (Supplementary Table S6a). GO term enrichment using topGO [82] identified 19 significantly enriched biological processes in *E. marisrubri* 6c exposed to host tissue extract (Fig. 3A; Supplementary Table S7a).

Proteome analyses detected 1972 proteins in *E*. *marisrubri* 6c across control and host tissue-treated samples. Of these, 14 were found to be significantly differentially abundant (0.7% of the proteome; FDR < 0.05, |FC| ≥ 0.5). A total of 11 proteins showed significantly higher abundance and two showed lower abundance (0.6 and 0.1% of the proteome, respectively; Supplementary Table S6b). Overall, TopGO identified 13 significantly enriched GO terms associated with exposure to host tissue extracts (Fig. 3B; Supplementary Table S7b).

Of note, the overlapping fraction between transcriptomic and proteomic datasets contained 1676 genes. About half (817) exhibited the same direction of change in both datasets. Of these, 473 exhibited up regulation and 344 downregulation. GO term enrichment analysis identified only three processes that were significantly upregulated in both transcriptome and proteome datasets, most notably ‘isopentenyl diphosphate biosynthesis’. This process encompasses the synthesis of isoprenoids, which includes multiple vitamins. Consistently downregulated were processes pertaining to protein/ribosomal function (Supplementary Table S8).

### Differential expression of genes implicated in cell-cell signaling and host-symbiont recognition

Broadly, the transcriptome response of *E. marisrubri* 6c exposed to coral host tissue extracts pertained to two distinct groups: DEGs and associated GO terms were associated with either cell-cell signaling and host-symbiont recognition or genes associated with cellular metabolism. Additional features are briefly discussed in the Supplementary Results and Discussion.

We found downregulation of motility functions, specifically the flagellar export and assembly genes *fliR*, *flhA*, *fliH*, *fliO*, which are part of the flagellar type III secretion apparatus. Reduced expression of flagellar assembly genes or flagellar structural modification is a common response of bacteria to settlement and colonization, although it can also be related to the evasion of the host’s immune defenses following exposure to holobiont cues or the successful infection of host cells [93, 94]. The observed downregulation of flagellar assembly genes in the present study could hence constitute one strategy by which *E. marisrubri* 6c facilitates colonization of its host.

**Fig. 4:**
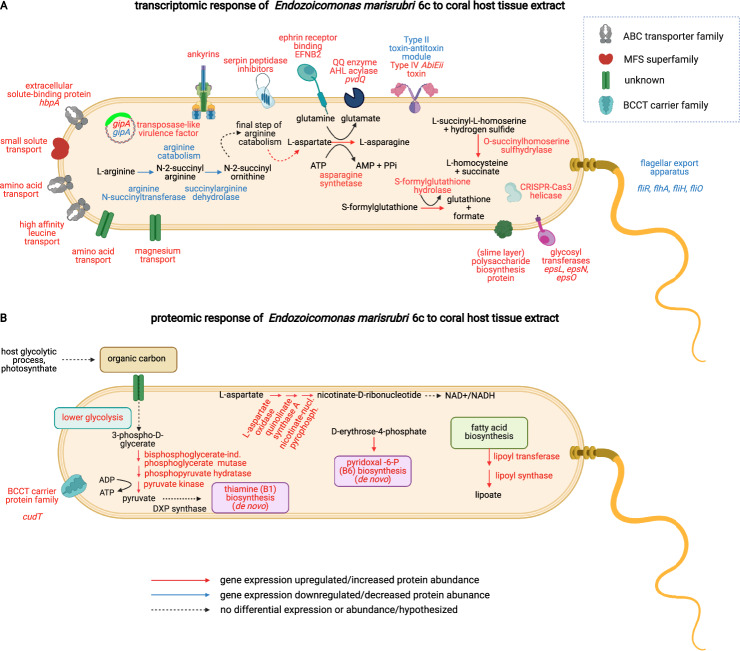
Reconstructed metabolic pathways and proteins in *Endozoicomonas marisrubri* 6c based on transcriptomic and proteomic responses of the bacterial cells to ultra-fractionated coral host tissue extract. **A** Regulation of differential gene expression within selected significant biological processes (GO terms) and significant transcripts (*p* < 0.05, |FC| = 2). **B** Regulation of proteins within selected significant biological processes (GO terms) and differentially abundant proteins (adjusted *p* < 0.05, |FC| = 0.5) in the *E. marisrubri* 6c proteome.

Other features suggesting host response modulation by *E*. *marisrubri* 6c included the upregulation of ankyrin repeats (DESeq2, FC ≥ 2, adjusted *p* < 0.05; Supplementary Table S6a; Fig. 4A). Ankyrins are eukaryote-like proteins that mediate protein-protein interactions in biological processes pertaining to an intracellular lifestyle, and hence suggested modulators of eukaryote-prokaryote interactions [95, 96], and their genomic abundance has previously been associated with coral bacteria in symbiosis [33, 37]. Ankyrin expression in recombinant *E*. *coli* cells has been shown to inhibit phagocytosis by amoebal cells in sponges via phagosomal arrest, resulting in the accumulation of bacteria in the sponge phagosome [97]. Together, the presence and diversity of CDSs encoding putative ankyrin repeats across *Endozoicomonas* genomes ([33]; Fig. 2B), including the genome of *E*. *marisrubri* 6c, and the upregulation of putative ankyrins by *E*. *marisrubri* 6c in response to holobiont cues (coral host tissue extract) here, may not only help explain the high prevalence of *Endozoicomonas* in coral tissues [31] but may also suggest that similar mechanisms are involved in the establishment of coral–*Endozoicomonas* symbioses.

GO term enrichment analysis suggested further potential mechanisms associated with host modulation upon exposure to *E*. *marisrubri* 6c. These included GO terms associated with ‘protein modification in other organism’, ‘regulation of chemotaxis’, ‘negative regulation of endopeptidase activity’, and ‘regulation of GTPase activity’ (topGO: KS test, *p* values < 0.05; Fig. 3A; Supplementary Table S7a). Further query of these GO terms revealed the differential expression of ephrin receptor binding domain EFNB2 (associated with ‘regulation of chemotaxis’) and multiple serine protease inhibitors (serpins; associated with ‘negative regulation of endopeptidase activity’ and ‘regulation of chemotaxis’), as well as ribonucleotide reductases (‘protein modification in other organism’; ‘regulation of GTPase activity’) (Fig. 4A; Supplementary Table S7a).

EFNB2 ephrin receptor binding domains (‘ephrins’) are eukaryote-like proteins containing ephrin ectodomains and secretion signals at their N-terminus, and were previously reported to be present in the genome of *E*. *montiporae* [14, 98]. Ephrins act as signal molecules in animals [99], and their binding to ephrin receptors activates various intracellular signaling pathways, including endocytosis [100]. EFNB2 in particular has been proposed to play a role in targeting host ephrin receptors to initiate internalization, i.e., invasion of the host cell via endocytosis [14]. Serpins on the other hand have been suggested to inhibit host serine proteases and peptidases [101] and may facilitate infection by avoiding digestion [102]. Some serpins associated with human gut bacteria are carbohydrate-regulated, supposedly via catabolite repression [101], a mechanism that could be relevant for activation of bacterial serpins upon infection of the symbiotic coral holobiont, where organic carbon (e.g., in the form of photosynthate) is plentiful [103]. In summary, *E. marisrubri* 6c may employ a complex, orchestrated cascade of mechanisms for successful infection and colonization of its host by modulating phagosomal processes and preventing digestion. Importantly, while the discussed features (ankyrins, ephrins, and serpins) have been primarily investigated in intracellular bacteria, at present we do not know the exact location of *E*. *marisrubri* 6c in the coral host. Characterization of their particular niche within the intact symbiosis will help further elucidate their roles, functionality, and interactome in the coral holobiont.

To assess whether the proposed model of infection may be universal for *Endozoicomonas* originating from different animal hosts, we compared abundances of gene families (gene copies *per* Mbp) across genomes. We found that ankyrins are widely distributed and present across all analyzed *Endozoicomonas* genomes, but copy numbers (*per* Mbp) vary greatly. On average, ankyrins were more diverse in clade A (Fig. 2B) than in clade B, in which *E*. *marisrubri* 6c is placed. *E*. *marisrubri* 6c carries a disproportionately high number of gene copies of the ankyrin repeat Ank_4 *per* Mbp compared to the other genomes. Serpins are present in *Endozoicomonas* genomes of both clades, while ephrins are present only in the genomes of coral-associated *Endozoicomonas* of clade B; specifically, *E*. *marisrubri* 6c, *E*. ‘humilis’, and *E. montiporae* (Fig. 2B). This suggests that ankyrins may be a universal feature of *Endozoicomonas* genomes, whereas serpins and ephrins may be more host-, clade-, or species-specific. While further work is required, this observation not only highlights marked differences in the genome of *E*. *marisrubri* 6c compared to those of other *Endozoicomonas* but may potentially suggest differences in the establishment of the host-symbiont relationship.

Finally, to assess whether DMSP catabolism is a universal feature of *Endozoicomonas* in marine holobionts, we performed a comparative approach using multiple annotation tools (Prokka, KEGG, UniProt) to assess the distribution of genes associated with DMSP catabolism across the *Endozoicomonas* genomes. DMSP demethylase *dmdA*, which catalyzes the first step of the DMSP demethylation pathway [104], was not found in any of the *Endozoicomonas* genomes that were screened. The DMSP lyases *dddD* and *dddY* were found in the genomes of *E*. *acroporae* and *E*. ‘pistillata’ Type B, respectively. Both genes catalyze distinct initial biotransformation steps in the DMSP cleavage pathway, resulting in the production of 3-hydroxypropionate and acrylate, respectively, from DMSP. Neither genes were found in the genomes of *E*. *marisrubri* 6c or those of other *Endozoicomonas* (Fig. 2C), lending support to previous findings by [32] who reported the presence of *dddD* only, and only for *E*. *acroporae*. Our findings thereby suggest that, while DMSP degradation may be an important metabolic trait in marine bacterial symbioses [19, 32], it is not a universal feature among *Endozoicomonas*.

### Transcriptional changes of genes implicated in amino acid metabolism

Transcriptional responses of *E. marisrubri* 6c cells to holobiont cues associated with metabolism included the upregulation of high affinity branched amino acid and leucine transporters (DESeq2; *p* value < 0.05; LFC ≥ 2). Further, different processes associated with amino acid metabolism (the final steps of asparagine synthesis and L-homocysteine formation), as well as features associated with polysaccharide (slime layer) biosynthesis, prokaryotic extracellular solute-binding proteins (*opuAC*), and small solute transport were significantly upregulated, while arginine catabolic processes were downregulated (Supplementary Table S6a; Fig. 4A).

The differential expression of genes associated with amino acid metabolism suggests that *E. marisrubri* 6c may have responded to amino acids and their precursors in the host tissue extract (refer to Fig. 4A and Supplementary Table S6a). While further studies *in hospite* are required, this suggests that *E. marisrubri* 6c may be able to respond to changes in holobiont amino acid availability. Amino acids contribute to a “currency” of interactions within a holobiont regulated by nitrogen limitation [18, 105–107]. For instance, Symbiodiniaceae may translocate a fraction of the amino acids they metabolize to the host [108–112]. Further, bacteria have been proposed as sources and sinks of amino acids within the coral holobiont [13, 37, 86], and use amino acids as cues to locate and “home in” on a suitable host with which to establish symbiosis [113, 114].

### Proteomic response of *E. marisrubri* 6c to coral holobiont cues suggest metabolic cross-talk *in hospite*

Processes related to the biosynthesis of vitamins and other cofactors, as well as energy metabolism, were significantly enriched in the *E*. *marisrubri* 6c proteome (Supplementary Results and Discussion). The most significant GO term in the proteomic response to holobiont cues was ‘thiamine (vitamin B1) biosynthetic process’ (KS test, *p* value = 0.0076) (Figs. 3B, 4B). In addition, the GO terms ‘pyridoxal phosphate (vitamin B6) biosynthetic process’ and ‘lipoate biosynthesis’ were enriched (KS test *p* values = 0.0362 and 0.0221, respectively; Figs. 3B; 4B; Supplementary Fig. S4). Features in GO terms associated with the biosynthesis of both B vitamins and the cofactor lipoate were upregulated (Fig. 3B). In addition, GO terms associated with energy metabolism were significantly upregulated, including ‘glycolytic processes’, ‘*de novo* NAD biosynthetic process from aspartate’, ‘NADH metabolic process’, and ‘quinolinate biosynthetic processes’ (Supplementary Tables S6b, S7b; Supplementary Fig. S4).

The increase in abundance of proteins related to B vitamin biosynthesis by *E. marisrubri* 6c in response to host tissue extract is of particular interest. Animals and most algae, including dinoflagellates, are auxotrophic for B vitamins, and must therefore acquire them from their diet or bacterial symbionts [37, 115–119]. *Endozoicomonas*, including *E*. *marisrubri* 6c, harbor biosynthetic gene clusters for different B vitamins [13, 14], and the clusters for vitamin B1 and B6 biosynthesis are present across all screened genomes (Fig. 2B). Therefore, it may well be possible that *Endozoicomonas* contribute to both the coral host’s and algal symbionts’ metabolic requirement for B vitamins, which in the specific case of *E*. *marisrubri* 6c includes vitamins B1, B6, and potentially B7 (as reflected in biotin synthase *bioB* protein abundance trending upwards in the proteome; Supplementary Results and Discussion, Supplementary Table S6b). These B vitamins are essential coenzymes involved in basic cellular processes. These include energy production and central metabolism, in particular carbon assimilation, respiration, and primary carbohydrate metabolism (vitamin B1), amino acid metabolism (vitamin B6), carboxylases involved in fatty acid biosynthesis, gluconeogenesis, amino acid and fatty acid degradation (vitamin B7), and osmolyte and antioxidant production (vitamin B1) [118, 120, 121]. Vitamin B1 is known as a component of stress responses of autotrophs, in particular in the context of plant disease resistance, stress tolerance, and crop yield [120].

In this study we cannot currently quantify vitamin B production, discriminate whether *E. marisrubri* 6c (or other *Endozoicomonas*) channels its entire vitamin B pool into its own metabolic processes, or whether translocation to the host and/or algal symbiont compartment occurs, and if so, to a physiologically significant extent. Assuming translocation of B vitamins does indeed occur, reductions in *Endozoicomonas* abundance, as commonly observed in stressed, diseased, and bleached corals [9, 27–30, 122], would therefore translate into a reduced supply of these essential coenzymes, and hence, compromised stress tolerance. Reduced *Endozoicomonas* abundances could thereby further exacerbate the overall health of an already compromised holobiont.

The query of features assorted under ‘glycolytic processes’ identified an increased abundance of proteins associated with the lower glycolytic or trunk pathway, which encompasses the final conversions from 3-phospho-D-glycerate to pyruvate. Within the holobiont, *E. marisrubri* 6c could potentially obtain 3-phospho-D-glycerate either from glycolytic processes of the host or from algal photosynthesis (3-phospho-D-glycerate constitutes the final product of carbon fixation in the C3 pathway of photosynthesis; [123]). The increased abundance of proteins associated with lower glycolysis thereby likely reflects the overall higher organic carbon availability in coral host tissue extract compared to seawater.

### Holobiont cues prime *Endozoicomonas* for a symbiotic lifestyle

Despite a large and increasing number of studies characterizing coral-associated prokaryotic community assemblages and dynamics, in addition to emerging evidence that the microbiome is a key factor contributing to host health, stress tolerance, and resilience [124, 125], we still lack a basic understanding of the molecular processes that drive recognition, setup, and maintenance of coral-prokaryote interactions. Here we sought to explore the molecular responses underlying the association of the coral *A. humilis* with its bacterial symbiont *E. marisrubri* 6c. To do this, we employed a multi-faceted approach where we first obtained a bacterial isolate and characterized its genome, which facilitated subsequent functional gene expression and proteome profiling on the host tissue extract-exposed bacterial isolate. Approaches combining culture-dependent with -independent applications are still rare, but critical to advance insight into the molecular underpinnings of coral-prokaryote interactions. We stress that putative processes identified using this approach will still require *in hospite* validation, i.e. in the intact symbiosis.

*Endozoicomonas* genomes are large and characterized by a diversity of gene clusters for the metabolism and biosynthesis of amino acids, vitamins, and cofactors [9, 13, 14], and the novel *E*. *marisrubri* 6c described in the present study is no exception. While previous reconstructions of potential host-*Endozoicomonas* interactions have been hypothesized based on (meta)genomic evidence, here we provide the first assessment of transcriptomic and proteomic responses of *E. marisrubri* 6c in response to holobiont cues in vitro. Overall, these responses suggest that holobiont-derived cues induce several behavioral, physiological, and metabolic changes which may prime bacterial associates for switching to a symbiotic lifestyle.

**Fig. 5:**
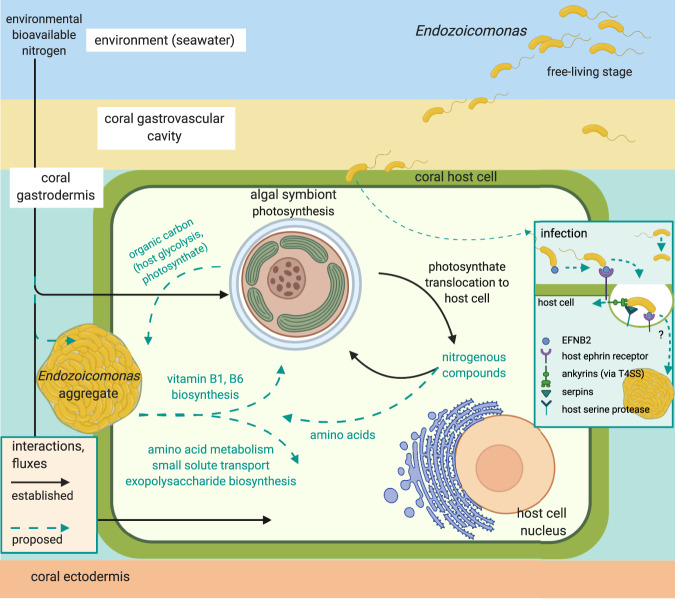
Proposed interactions involved in symbiosis establishment and maintenance in the *Acropora*-Symbiodiniaceae-*Endozoicomonas* system based on transcriptomic and proteomic responses of *E. marisrubri* 6c to coral holobiont cues.

Gene expression changes suggest that *E. marisrubri* 6c may have the ability to home in on suitable hosts via motility and chemotaxis, e.g., by sensing holobiont-derived metabolites [113, 114] (Fig. 5). Upon encountering its coral host, *E. marisrubri* 6c may then initiate a cascade of mechanisms to evade or modulate host immune responses: (a) the downregulation of expression of flagellar assembly genes, potentially accompanied by flagellar restructuring [93]; (b) ephrin receptor binding by EFNB2 to initiate internalization via phagocytosis [14, 98, 100]; and (c) co-expression of ankyrins and serpins to induce early phagosomal arrest [95, 97] and to directly interfere with digestion by inhibiting host proteases and peptidases [101, 102], respectively.

Following cellular internalization and subsequent invasion of its site of symbiosis within the host system, *E. marisrubri* 6c may subsequently proliferate and form aggregates in the coral tissues, in close proximity to where the Symbiodiniaceae reside [9, 13] (Fig. 5). Our findings suggest that in the intact symbiosis, interactions between *E. marisrubri* 6c, the coral host, and the Symbiodiniaceae may include, but are not necessarily limited to: (a) amino acid metabolism; (b) biosynthesis and provisioning of essential B vitamins; and (c) utilization of organic carbon sources (stemming from e.g., products of host glycolysis, photosynthates) by *E. marisrubri* 6c. Importantly, the processes potentially implicated in symbiotic establishment and maintenance as proposed here may not be exclusive to associations with reef-building corals. Provisioning of essential metabolites, and B vitamins in particular, could help explain the prevalence of *Endozoicomonas* in forming symbiotic relationships with a range of distantly related marine animal hosts, such as corals, sponges, or ascidians [26].

Importantly, while the here-characterized *E*. *marisrubri* 6c occurs at low relative abundances in its native host, rare taxa in microbial communities can have an important and over-proportionate role in biogeochemical cycles, and consequently, abundance is not a *sensu stricto* indicator of functional importance [126]. It is hypothesized that rare *Endozoicomonas* may belong to (a) ubiquitous and metabolically relevant functional group(s) in coral holobionts comparable to the widely studied diazotrophs, i.e., dinitrogen-fixing prokaryotes [11, 127]. In the present study we found that biosynthetic gene clusters for vitamins B1 and B6 are present across all screened *Endozoicomonas* genomes, suggesting that B vitamin metabolism is widely shared within this bacterial genus, even though different *Endozoicomonas* are otherwise metabolically distinct. It is therefore likely that certain putative bacterial contributions, such as essential metabolite supply within the holobiont, may be derived from multiple co-occurring taxa, including different *Endozoicomonas*.

## Conclusion

Our work highlights the importance of obtaining bacterial isolates for functional studies of marine host-microbe systems. Here, by combining cultivation-dependent techniques with -omics applications, we shed light on the potential functions and interactions of the novel *E. marisrubri* 6c in its native coral host *A*. *humilis*. We show that *E. marisrubri* 6c not only responds to coral holobiont cues but that transcriptomic and proteomic data characterize several aspects of this response, including features related to modulation of the host immune response as well as changes in the metabolism. We propose that these responses resemble a behavioral, physiological, and metabolic priming of *E. marisrubri* 6c for a symbiotic lifestyle within the coral holobiont, where the bacterium may convey direct or indirect benefits to its host and associated algal symbionts via the provisioning of essential metabolites. The observed responses may in part explain the widespread association of *Endozoicomonas* with marine animals. Further ground-truthing of these results in the intact (coral) symbiosis is required to draw more definitive conclusions about the function(s) of *Endozoicomonas*. Functional studies to understand the drivers of metabolic cross-talk underlying the maintenance and dysbiosis of the coral–*Endozoicomonas* association should aim for a multi-faceted approach, for instance by combining microbiome manipulations and in-depth phenotyping applications with in vitro and *in hospite* sequencing, as well as metabolomics, imaging, and nanoscale secondary ion mass spectrometry (NanoSIMS) techniques. Other research directions could include the application of targeted functional gene knockouts in recombinant *Endozoicomonas* to investigate the here-proposed proposed model of infection and metabolite exchange or the assessment of host epigenetic responses to its bacterial symbiont.

## Supplementary information

Supplementary Material

Supplementary Table S1

Supplementary Table S2

Supplementary Table S3

Supplementary Table S4

Supplementary Table S5

Supplementary Table S6a

Supplementary Table S6b

Supplementary Table S7

Supplementary Table S8

## Data Availability

The annotated bacterial genome assembly is available on RAST (genome ID 6666666.314155; login credentials: Username: guest; Password: guest). Determined sequencing data (16S rRNA gene sequences and RNA-Seq) are available on NCBI under BioProject PRJNA753662. The mass spectrometry proteomics data are available via the PRIDE partner repository with the dataset identifier PXD027178 and DOI 10.6019/PXD027178. The bacterial type strain of *Endozoicomonas* 6c will become available at the German Collection of Microorganisms and Cell Cultures (DSMZ) in Braunschweig, Germany.
