# Supplementary material for: Coral holobiont cues prime Endozoicomonas for a symbiotic lifestyle
Source: ISME J. 2022 Apr 20;16(8):1883–95. doi: 10.1038/s41396-022-01226-7 (PMC9296628; doi:10.1038/s41396-022-01226-7)
Supplement: Supplementary file 1 — Supplementary Material [file 41396_2022_1226_MOESM1_ESM.pdf]

Coral holobiont cues prime *Endozoicomonas* for a symbiotic lifestyle

Claudia Pogoreutz[1,2], Clinton A. Oakley[3], Nils Rädecker[1,2,4], Anny Cárdenas[1,2], Gabriela Perna[1,2], Nan Xiang[1,5,6], Lifeng Peng[3], Simon K. Davy[3], David K. Ngugi[1,7], Christian R. Voolstra[1,2]

[1]Department of Biology, University of Konstanz, 78457 Konstanz, Germany

[2]Red Sea Research Center, Biological, Environmental, Science and Engineering, King Abdullah University of Science and Technology (KAUST), Thuwal 23955, Saudi Arabia

[3]School of Biological Sciences, Victoria University of Wellington, Wellington 6012, New Zealand

[4]Laboratory for Biological Geochemistry, School of Architecture, Civil and Environmental Engineering, École Polytechnique Fédérale de Lausanne (EPFL), 1015 Lausanne, Switzerland

[5]Alfred-Wegener Institute, Helmholtz Centre for Polar and Marine Research, 27570 Bremerhaven, Germany

[6]Marine Ecology Department, Faculty of Biology and Chemistry, University of Bremen, 28359 Bremen, Germany

[7]Leibniz Institute DSMZ – German Collection of Microorganisms and Cell Cultures GmbH, 38124 Braunschweig, Germany

Supplementary information

Supplementary Results and Discussion

<u>Gene expression changes by *Endozoicomonas marisrubri* 6c suggest responses to other bacteria and viruses in the holobiont</u>

Further features associated with responses to other bacteria as well as to viruses were significantly differentially regulated. These include the upregulation of the quorum quenching enzyme AHL acylase *pvdQ* as well as a phage integrase family gene, the head morphogenesis protein SPP1 gp7, CRISPR-associated helicase Cas3 (DESeq2; p value < 0.05; FC ≥ 2); as well as the downregulation of viral recombinase domains (DESeq2; p value < 0.05; FC ≥ -2; Supplementary Table 6a,b). Differential regulation of these features suggests responses of *E. marisrubri* 6c to the presence of metabolites and/or nucleic acid fragments of holobiont-associated bacteria (e.g., *via* upregulation of AHL acylase; but see also [1] who suggest *Pseudomonas aeruginosa* express this enzyme to modulate its own quorum-sensing-dependent pathogenic potential upon infection) and viruses (e.g., CRISPR-Cas3, part of the prokaryotic adaptive immune system against foreign nucleic acids; [2] in the host extract that may have passed through in the tissue fractionation process. While the differential expression of these features in response

to host cues *in vitro* may not necessarily represent natural responses of *E. marisrubri* 6c in the intact holobiont, it suggests it may readily respond to potential competitors and viruses.

Proteomic response of *E. marisrubri* 6c to holobiont cues

The most strongly upregulated protein in the *E. marisrubri* 6c proteome in the presence of host tissue extracts was a betaine-carnitine-choline transporter (BCCT carrier) (FDR, ~1.7 FC increase, adjusted p value < 0.05). The observed upregulation of glycine betaine transporters as well as small solute binding proteins (*opuAC*; cf. transcriptomic response in the main manuscript; Figure 4) in response to holobiont cues aligns well with previous reports of high gene expression of pathways involved in glycine-betaine metabolism in symbiotic and aposymbiotic Cnidaria, across larval and adult stages, and in symbiotic Symbiodiniaceae [3–5]. Corresponding upregulation of BCCT carriers in *E. marisrubri* 6c in response to host extracts lends support to the notion that symbiotic bacteria may contribute to shaping intracellular pools of glycine betaine within the coral holobiont and as such may be drivers of osmoregulation and nitrogen cycling [5]. Finally, another notable feature in the proteome is biotin synthase *bioB*, which is exhibiting a trend towards upregulation (Supplementary Table 6b). Biotin is another essential cofactor (Vitamin B7), and thereby of potential relevance for the coral host and Symbiodiniaceae.

Supplementary results: Figures and table captions

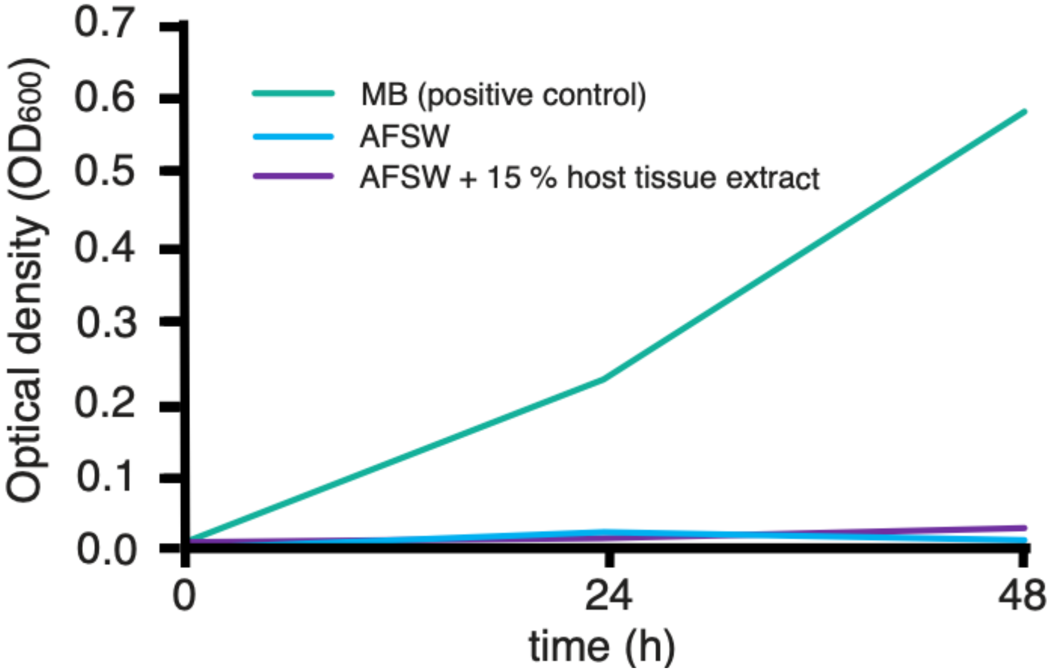

Supplementary Figure S1. Optical density measurements (OD_{600}) of different media inoculated with *Endozoicomonas marisrubri* 6c cells, specifically autoclaved, filtered seawater (AFSW), AFSW + 15 % host tissue extract, and standard Difco2216 Marine Broth (positive control) at 28 °C over the course of 48 h. No growth was obvious from *E. marisrubri* 6c cells in AFSW and AFSW + 15 % host tissue extract.

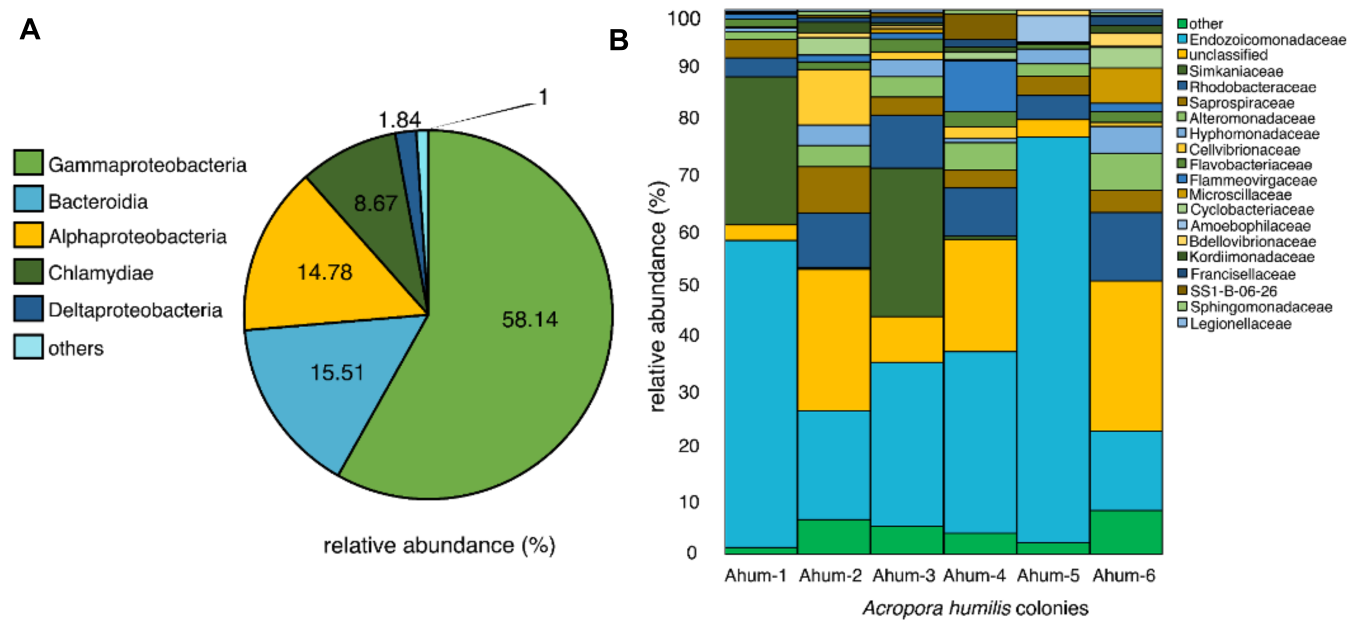

Supplementary Figure S2. Bacterial community composition associated with the tissues of the common Red Sea coral, *Acropora humilis* (n = 6). **A**. Relative abundances of most abundant bacterial classes based on 16S rRNA gene sequencing. **B**. Relative abundances of 15 most abundant bacterial families based on 16S rRNA gene sequencing.

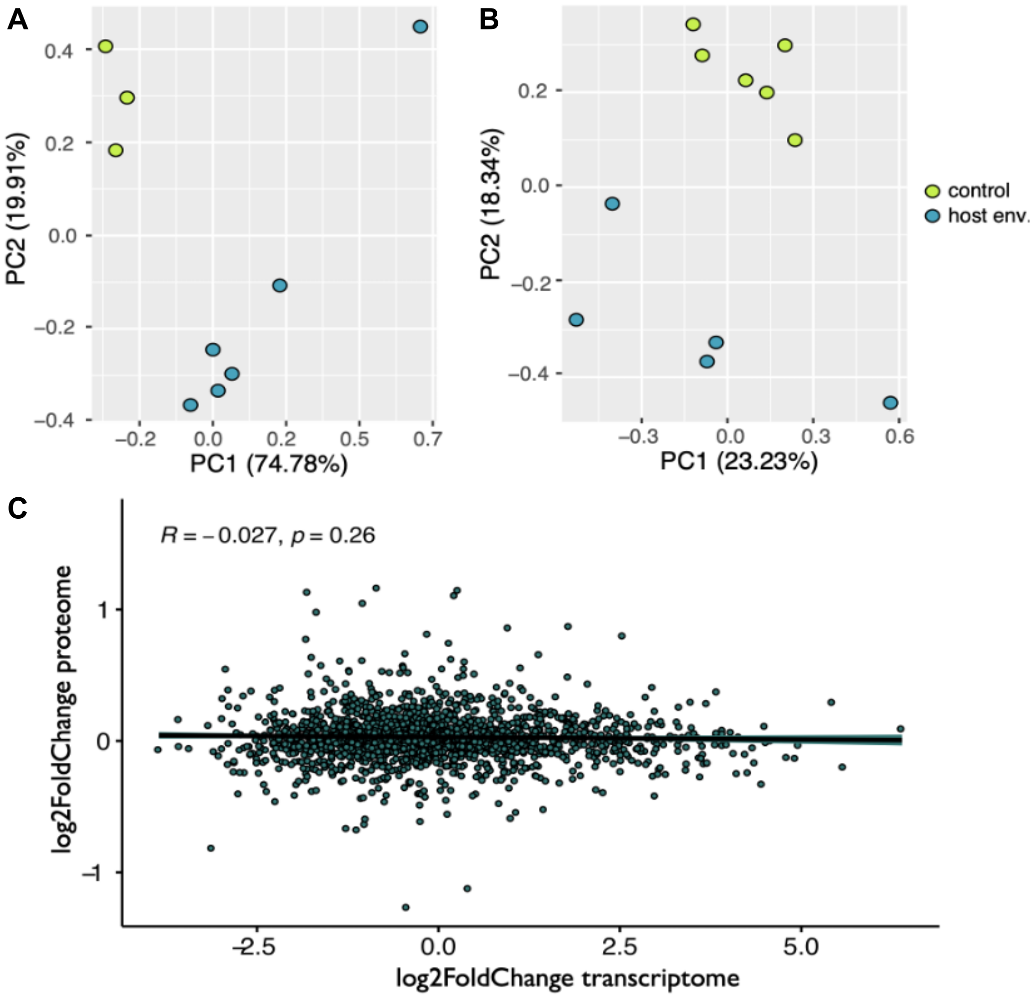

Supplementary Figure S3. Transcriptomic and proteomic responses of *Endozoicomonas marisrubri* 6c after 3h exposure to coral host tissue extract. **A**. Principal component analysis (PCA) plot of transcriptomic response; **B**. PCA plot of proteomic response; **C**. Pearson correlation plot of $\log_2$ fold changes (FC) in expression or abundance of transcripts and proteins, respectively. Only overlapping genes and proteins, i.e., present in both datasets were considered. No correlation of FC between transcriptomic and proteomic response was observed.

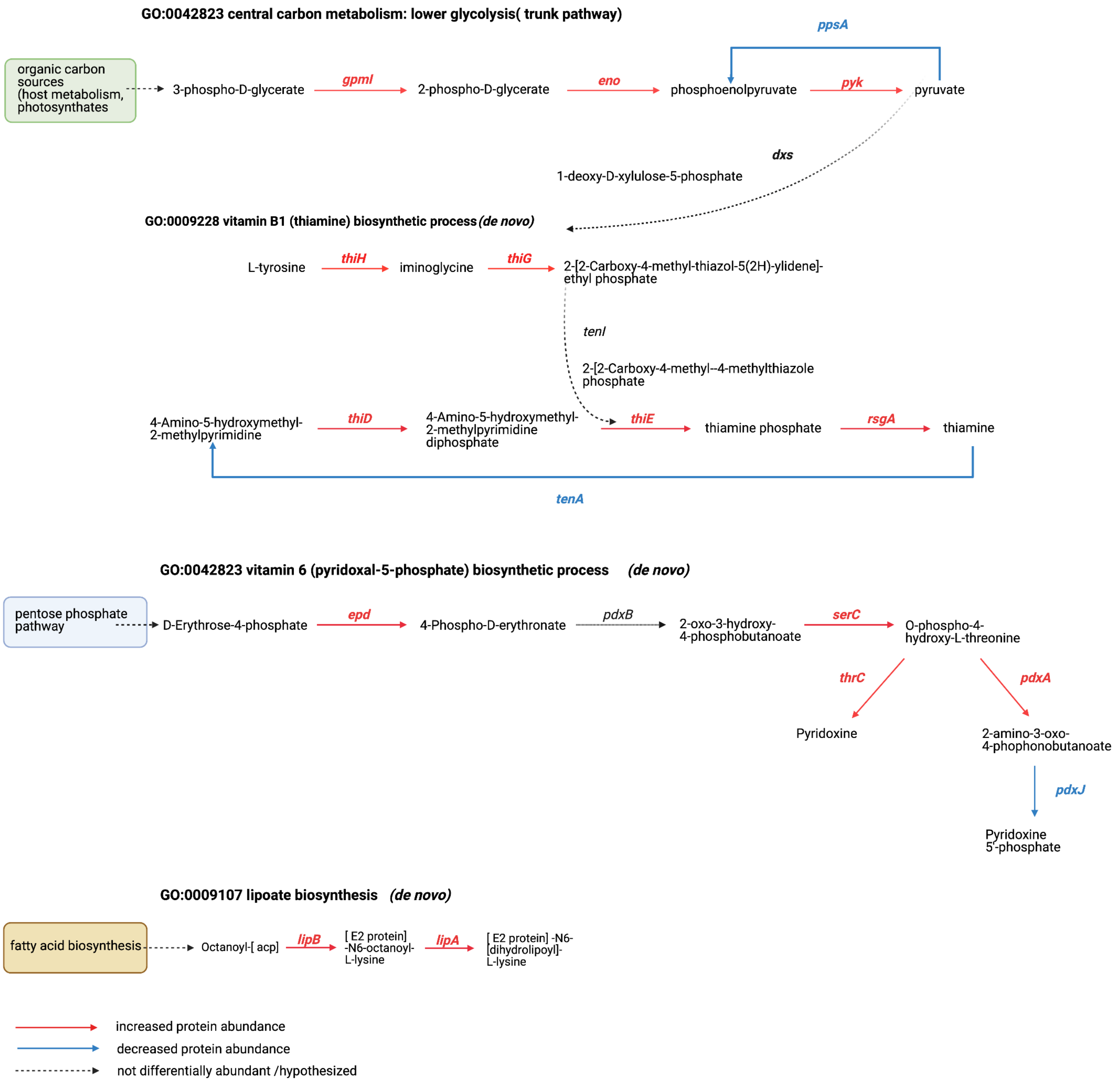

Supplementary Figure S4. Reconstructed metabolic pathways and direction of change of individual proteins within selected significant biological processes (GO terms) in the *Endozoicomonas marisrubri* 6c proteome in response to coral host tissue extract as identified with GO term enrichment analysis.

Supplementary Tables are provided separately.

Supplementary Table S1. Bacterial ASV count table, taxonomic annotation of bacterial community composition associated with the tissues of the common Red Sea coral *Acropora humilis*.

Supplementary Table S2. Absolute quantification of *Endozoicomonas marisrubri* 6c in the total tissue-associated bacterial microbiome of the common Red Sea coral *Acropora humilis via* qPCR using universal bacterial and *E. marisrubri* 6c-specific primer pairs. The abundance of *E. marisrubri* 6c is expressed as percentage of gene copy numbers of the total 16S rRNA gene copy numbers.

Supplementary Table S3. Detailed breakdown of annotated features (genes) distributed over SEED Subsystems (as implemented in RAST) in the genome of the novel *Endozoicomonas marisrubri* 6c.

Supplementary Table S4. Results of GGDC (formula d_4, i.e., calculations of DDH and distance calculations based on identities / HSP length) using the draft genome of *Endozoicomonas marisrubri* 6c as a query genome.

Supplementary Table S5. Average Nucleotide Identity (ANI) and Amino Acid Identity (AAI) values of novel *Endozoicomonas marisrubri* 6c compared to other available *Endozoicomonas* nucleotide and amino acid sequences. All ANI and AAI values are << 95. SeqA = Identity of query genome. SeqB = Identity of reference genome. SD = standard deviation of identity between reciprocal best-matching fragments. N = number of reciprocal best matches found. Omega = minimum number of fragments (ANI) or proteins (AAI) between the genomes. Frx = N/Omega ratio (%), i.e., the percentage of the genome shared.

Supplementary Table S6. Full list of differential features in the response of *Endozoicomonas marisrubri* 6c in response to coral host tissue extract (relative to control). a) Differentially expressed genes as identified by DESeq2 (statistical significance: $p < 0.05$; $|FC| > 2.0$). b) Differentially abundant proteins as identified by the limma algorithm (FDR; statistical significance: $p < 0.05$; $|FC| > 0.5$).

Supplementary Table S7. List of significant biological processes based on Gene Ontology (GO) term enrichment characterizing the response of *Endozoicomonas marisrubri* 6c to coral host cues (relative to seawater; KS test). a) Significant GO terms based on gene expression changes. b) Significant GO terms based on changes in protein abundances.

Supplementary Table S8. List of significant biological processes based on Gene Ontology (GO) term enrichment characterizing overlapping and consistently regulated responses (up- or downregulated) of *Endozoicomonas marisrubri* 6c to coral host cues (relative to not consistently regulated responses; Fisher's test) in both transcriptomic and proteomic datasets.

Supplementary References

1. Sio CF, Otten LG, Cool RH, Diggle SP, Braun PG, Bos R, et al. Quorum quenching by an N-acyl-homoserine lactone acylase from *Pseudomonas aeruginosa* PAO1. *Infect Immun* 2006; **74**: 1673–1682.
2. Brouns SJJ, Jore MM, Lundgren M, Westra ER, Slijkhuis RJH, Snijders APL, et al. Small CRISPR RNAs guide antiviral defense in prokaryotes. *Science* 2008; **321**: 960–964.
3. Oakley CA, Ameismeier MF, Peng L, Weis VM, Grossman AR, Davy SK. Symbiosis induces widespread changes in the proteome of the model cnidarian Aiptasia. *Cell Microbiol* 2016; **18**: 1009–1023.
4. Sproles AE, Oakley CA, Matthews JL, Peng L, Owen JG, Grossman AR, et al. Proteomics quantifies protein expression changes in a model cnidarian colonised by a thermally tolerant but suboptimal symbiont. *ISME J* 2019; **13**: 2334–2345.
5. Ngugi DK, Ziegler M, Duarte CM, Voolstra CR. Genomic blueprint of glycine betaine metabolism in coral metaorganisms and their contribution to reef nitrogen budgets. *iScience* 2020; **23**: 101120.
